# No association between serum uric acid and lumbar spine bone mineral density in US adult males: a cross sectional study

**DOI:** 10.1038/s41598-021-95207-z

**Published:** 2021-08-02

**Authors:** Xiaoli Li, Lianju Li, Lixian Yang, Jiaxun Yang, Hua Lu

**Affiliations:** 1 Department of Rheumatology, Xingtai People’s Hospital, Xingtai, 054001 Hebei China; 2 Department of Internal Medicine, Hebei Medical University, Shijiazhuang, 050017 Hebei China; 3 Department of Breast Surgery, Xingtai People’s Hospital, Xingtai, 054001 Hebei China; 4 Department of Information Center, Xingtai People’s Hospital, Xingtai, 054001 Hebei China; 5 Department of Nephrology, Xingtai People’s Hospital, Xingtai, 054001 Hebei China

**Keywords:** Endocrinology, Health care, Rheumatology, Risk factors

## Abstract

Available evidence linking serum uric acid (SUA) and bone mineral density (BMD) remains controversial, and data on this association are limited among adult men in the general population. Thus, the aim of this study was to evaluate the association of SUA with lumbar spine BMD in US adult males. A cross-sectional study was conducted based on the National Health and Nutrition Examination Survey (NHANES, 1999–2006) database. Multivariate linear regression analyses were employed to assess the association of SUA with lumbar spine BMD, considering complex survey design and sampling weights. Through rigorous eligibility criteria, a total of 6704 individuals were yielded for final data analysis (average age, 40.5 years; 70.6% white). After fully adjusting potential confounders, no associations were detected between SUA and lumbar spine BMD [β (95% confidence interval, CI), − 0.003 (− 0.007, 0.002)]. Additionally, similar results were observed in all stratification analyses, and no interactions were found based on all priori specifications. In brief, our findings did not provide an inspiring clue for the hypothesis that SUA may be beneficial to lumbar spine BMD. Future more prospective studies are needed to further explore the causal relationship of SUA with lumbar spine BMD.

## Introduction

Osteoporosis has become a growing public health issue because of its high prevalence worldwide and heavy financial burden on individuals and society^1,2^. In particular, lumbar spine fractures almost require surgical fixation, and thereby result in greater suffering, larger economic costs, and higher short-term mortality risk^2–4^. Additionally, lumbar spine fractures, as prototypical osteoporotic fracture, were closely related to the reduction of lumbar bone mineral density (BMD)^5^. Thus, more risk factors for lowering BMD require to be identified and are vital for devising public health strategies.

Serum uric acid (SUA), a final product of purine metabolism^6^, represents the major risk factor for gouty arthritis and renal injury. Substantial evidence has demonstrated raised SUA levels are related to higher risk of various adverse outcomes, including diabetes mellitus (DM), metabolic syndrome, chronic kidney disease (CKD), cardiovascular disease and cancer^7–12^. However, some observational studies have reported SUA may be beneficial to BMD^13–16^, and one proposed mechanism may be via the potential anti-oxidant effect of uric acid, which prevents oxidative stress-related bone loss and osteoporosis^17^. However, whether UA actually exerts an antioxidant property in vivo is still controversial^18^, and existing studies of association between SUA and BMD have provided somewhat conflicting. The positive association of SUA with BMD was detected mostly in Asian population^13–15,19–21^, but not in American populations^22,23^. Even two studies^16,23^, both proofs from the National Health and Nutrition Examination Survey (NHANES), have reached different conclusions. More importantly, in most studies, some important factors affecting bone metabolism, including serum alkaline phosphatase (ALP), serum calcium, serum phosphorus, parathyroid hormone (PTH) and 25-OH-D (1,25D), were not fully adjusted as confounding factors^13,15,19–21^. Additionally, most studies exploring the relationship of SUA with lumbar BMD focused on peri- and post-menopausal women, or elderly men^13–16^, but this association in general adult men remains limited and equivocal.

To address this, we used data from the nationally representative NHANES (1999–2006) database, through rigorous inclusion criteria as well as fully adjusting confounding factors, to examine the association of SUA with lumbar spine BMD among US adult males.

## Methods

### Study design and population

NHANES is a nationally representative cross-sectional data providing the overall health and nutritional status of the civilian, non‐institutional US population. The design, data collection procedures, sample weight and informed consent have been described in detail at the National Center for Health Statistics, from which related data can be publicly available. For this cross-sectional study, the individuals for the analysis were screened from the NHANES 1999–2006 biennial surveys, which have been well integrated and spliced by Patel et al.^24^. Exclusion criteria included: women; men < 18 years old; missing SUA and BMD data; individuals with diagnosed DM, CKD (estimated glomerular filtration rate (eGFR) < 60 mL/min/1.73 m^2^), rheumatoid arthritis (RA) and malignancy, as well as those taking bisphosphonates, glucocorticoids, allopurinol, thiazide diuretics, sex hormones therapy, or thyroid replacement therapy. Finally, among 20,264 male participants, through strict eligibility criteria, a total of 6704 adult men with available SUA and lumbar spine BMD data were included in the study. All cycles of the NHANES protocols were approved by the institutional ethics review board of the National Center for Health Statistics^25^, and informed consent was obtained from each study participant before starting the survey. Besides, this study was conducted in compliance with the Strengthening the Reporting of Observational Studies in Epidemiology (STROBE) statement and the Declaration of Helsinki.

### Exposure variable and outcome variable

As the exposure variable of this study, SUA was measured using Roche Hitachi Model 917 or 704 Multichannel Analyzer between 1999 and 2001, and Beckman Synchron LX20 since 2002. As described in prior studies^26,27^, the coefficient of variation for SUA measurements in each cycle was approximately 2%, suggesting good repeatability. The outcome of interest was lumber spine BMD, which was measured by dual-energy X-ray absorptiometry (DXA) with a Hologic QDR-4500A fan-beam densitometer (Hologic, Inc., Bedford, Massachusetts). All measurements were performed by NHANES well-trained and certified radiology technologists.

### Covariates

The covariates, including sociodemographic variables, daily nutrient intake and blood biochemistry profile, were selected according to prior studies reporting risk factors for BMD. Questionnaire information was used to obtain gender, age, race/ethnicity (non‐Hispanic white, non‐Hispanic black, Mexican American, other race), physical activity (sedentary, low, moderate, high), education (< high school, high school, > high school), drinker (no or yes), and smoker (current, ever, never). Co-morbidities including DM, RA, CKD and malignancy were obtained by self-reported physician diagnosis. The drugs involved in this study included glucocorticoids, bisphosphonates, allopurinol, sex hormones therapy, thyroid replacement therapy, thiazide diuretics, and were obtained through prescription drug questionnaires that provided personal one-month prescription drug data before the survey. The dietary data collection was used to collect average daily nutrient intake estimation by a trained dietary interviewer, and dietary variables of interest included calcium supplementation, protein and energy intake. Key variables of body measurements involved weight, height, and body mass index (BMI) was calculated by dividing weight (kg) by height squared (m^2^). Routine blood biochemistry profile included albumin, urea nitrogen, creatinine, calcium, phosphorus, C-reactive protein (CRP), ALP, PTH, and 1,25D. Besides, eGFR was calculated using the Chronic Kidney Disease Epidemiology Collaboration equation^28^. Details of all variables can be publicly obtained at http://www.cdc.gov/nchs/nhanes/.

### Statistical analyses

We performed statistical analysis based on the CDC guidelines (https://wwwn.cdc.gov/nchs/nhanes/tutorials/default.aspx). We first dealt with missing data of covariates: for categorical variables (education, physical activity, drinking status and smoking status), missing data were regarded as an independent group^29^. For continuous variables, if missing data were small (< 5%), such as BMI, calcium supplementation, energy and protein intake, then the corresponding mean was used to supplement; and if missing data were large (> 20%), such as 1,25D and PTH, the dummy variables were employed to represent missing values^30^. Details of missing covariates were shown in Supplementary Table S1. Additionally, considering the complexity of survey design, sample weights were taken into consideration for statistical analysis according to the CDC guidelines. In the demographic files, the sample weight for 1999–2002 (WT99–02) was the variable WTMEC4YR of NHANES 1999–2000 and NHANES 2001–2002; and the weight for 2003–2004 (WT03–04) and 2005–2006 (WT05–06) were the variable WTMEC2YR of NHANES 2003–2004 and NHANES 2005–2006, respectively; then the sample weight for 1999–2006 (WT99–06) was calculated as WT99–06 = 0.5* WT99–02 + 0.25 * WT03–04 + 0.25 * WT05–06^31,32^.

The characteristics of the study population were presented as weighted means (standard error, Se) for continuous variables and weighted percentages (Se) for categorical variables. Weighted multivariate linear regression models were employed to assess the correlation of SUA with lumbar spine BMD in four distinct models. Model 1 was a non-adjusted model with no variable adjusted. Model 2 was adjusted for socidemograophic variables (age, race/ethnicity, education, physical activity, smoking and drinking). Model 3 was further adjusted for BMI, dietary intake factors (calcium supplementation, energy and protein intake) and blood biochemical variables (serum albumin, CRP, serum calcium, serum phosphorus, ALP, 1,25D, PTH, and eGFR). Model 4 was adjusted for variables according to a change in effect of more than 10% (age, race/ethnicity, smoking, BMI, serum albumin, eGFR, CRP, serum calcium, 1,25D and calcium supplementation). These confounders were selected on the basis of prior studies examining their associations with the outcomes of interest (model 3) or a change in effect of more than 10% (model 4). Additionally, serum ALP, PTH, and CRP were log2 transformed prior to regression analysis. To determine the robustness of results, sensitivity analyses were conducted. SUA was converted into categorical variables based on clinical cutoffs and quartiles, and then calculated the P for trend as taking the mean of SUA quartiles as the continuous variable. In addition, we further applied generalized additive model (GAM) to examine nonlinear or linear association of SUA with BMD using SUA concentration as a continuous variable in the fully adjusted model. If the non-linear relationship was observed, a two-piecewise linear regression model was conducted to calculate the threshold effect of the SUA on BMD in terms of the smoothing plot, and recursive method calculates automatically the inflection point, where the maximum model likelihood would be used. Subgroup analysis and interaction test were performed according to stratified by age, race/ethnicity, BMI, physical activity, education, drinking, smoking, eGFR and survey years. Interaction tests among subgroups were performed using the likelihood ratio test.

All statistical analyses were conducted using R statistics packages (http://www.R-project.org, The R Foundation) and EmpowerStats (http://www.empowerstats.com, X&Y Solutions, Inc, Boston, MA). *P* values less than 0.05 (two-sided) were considered statistically significant.

## Results

### Characteristics of the study population

**Figure 1:**
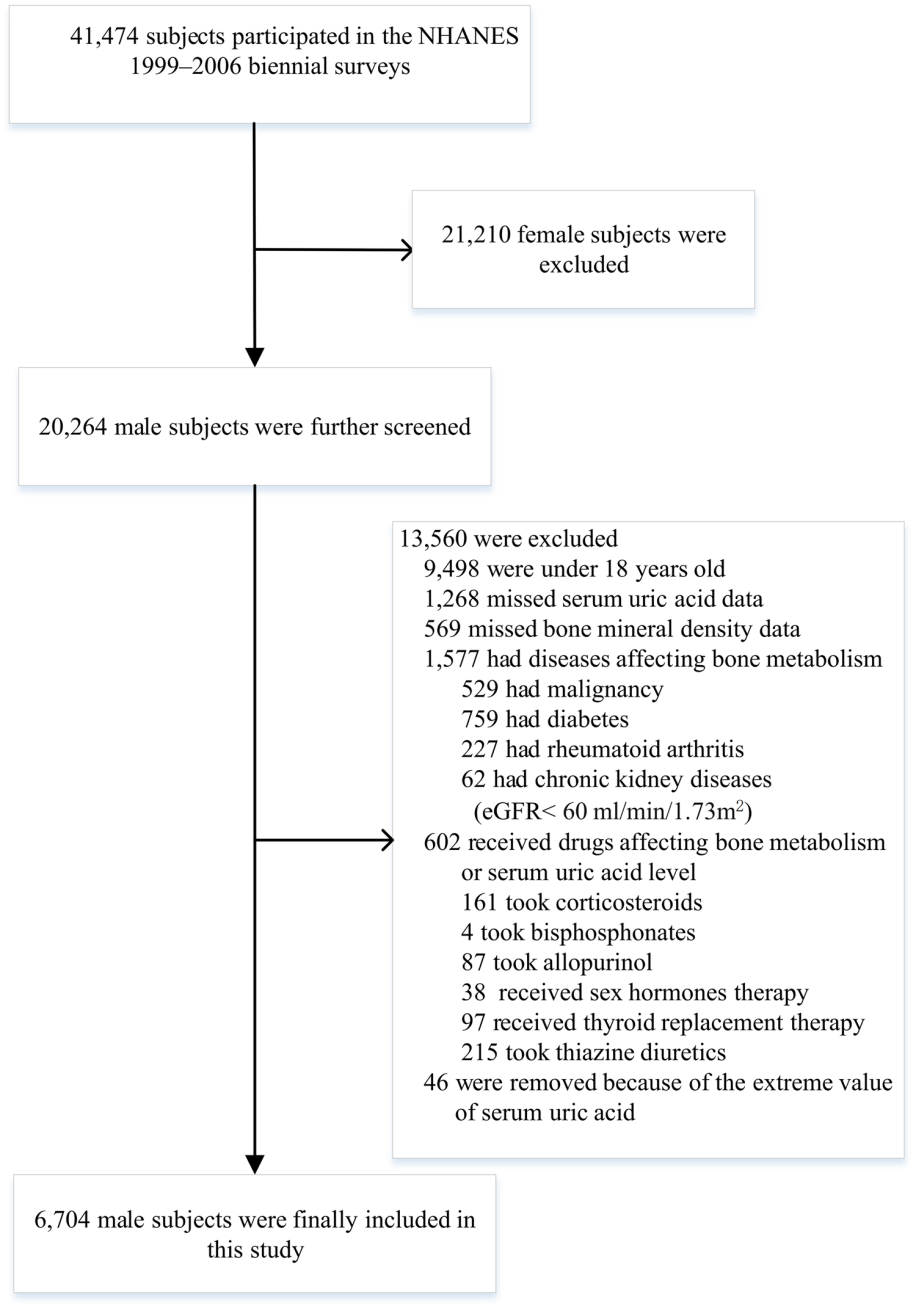
Selection of study participants in the National Health and Nutrition Examination Survey (NHANES, 1999–2006). eGFR estimated glomerular fltration rate.

A total of 6704 subjects aged 18–85 years were included in this study (NHANES1999–2000: 1595 participants; 2001–2002: 1781 participants; 2003–2004: 1742 participants; 2005–2006: 1586 participants). The detailed screening of study participants was presented in Fig. 1. Overall, the weighted mean age was 40.5 years old, 70.6% was non‐Hispanic white, and the average SUA level was 5.9 mg/dL. The weighted distributions of population characteristics according to SUA quartiles were demonstrated in Table 1 and Table 2. Significant differences were observed across all quartiles of SUA for age, race/ethnicity, weight, BMI, drinking and eGFR. Participants with the highest SUA in the top quartile (Q4) were more likely to be older, non-Hispanic white, non-drinkers, have a higher weight and BMI, or lower eGFR levels (*P* < 0.05). Besides, no significant differences were detected for lumber spine BMD across the SUA quartiles.

**Table 1:** Socidemograophic and dietary characteristics of study population based on Serum Uric Acid quartile, weighted.

Characteristics	Overall	Quartiles of serum uric acid (mg/dL)				
		Q1 < 5.2	Q2: 5.2–5.9	Q3: 6.0–6.7	Q4 > 6.7	*P* for trend
N, unweighted	6704	1536	1729	1623	1816	
Age, years	40.49 (0.27)	38.24 (0.53)	39.82 (0.42)	40.86 (0.49)	42.54 (0.42)	< 0.0001
Weight, kg	85.96 (0.30)	80.10 (0.55)	82.55 (0.44)	87.49 (0.53)	92.32 (0.62)	< 0.0001
Height, cm	176.45 (0.12)	175.96 (0.25)	176.17 (0.22)	176.67 (0.26)	176.89 (0.23)	0.001
Body mass index, kg/m^2^	27.55 (0.10)	25.81(0.16)	26.58 (0.14)	27.97 (0.16)	29.44 (0.18)	< 0.0001
**Race, %**						
Non‐Hispanic White	70.65 (1.41)	65.60 (1.85)	71.41 (1.60)	70.77 (1.75)	73.69 (1.61)	< 0.0001
Non‐Hispanic Black	10.05 (0.82)	12.08 (1.10)	9.11 (0.83)	9.93 (0.97)	9.48 (1.03)	0.03
Mexican American	9.26 (0.81)	10.32 (1.00)	9.35 (0.81)	8.85 (0.99)	8.72 (0.91)	0.02
Other race	10.04 (1.00)	12.00 (1.67)	10.13 (1.21)	10.45 (1.25)	8.11 (1.05)	0.01
**Education, %**						
< High school	19.52 (0.79)	20.10 (1.32)	19.12 (1.12)	17.45 (1.15)	21.32 (1.15)	0.52
High school	26.28 (0.86)	25.07 (1.58)	24.13 (1.29)	29.44 (1.57)	26.62 (1.38)	0.09
> High school	54.05 (1.15)	54.83 (1.86)	56.75 (1.47)	53.11 (1.84)	52.06 (1.59)	0.06
**Physical activity, %**						
Sedentary	12.94 (0.65)	13.83 (1.05)	10.91 (1.03)	13.04 (1.14)	14.08 (1.20)	0.45
Low	24.18 (0.71)	24.90 (1.33)	22.69 (1.25)	23.54 (1.22)	25.58 (1.27)	0.47
Moderate	17.94 (0.64)	19.67 (1.43)	18.56 (1.11)	16.94 (1.24)	16.92 (1.34)	0.09
High	34.73 (0.78)	31.99 (1.43)	36.98 (1.52)	35.90 (1.52)	33.69 (1.34)	0.72
**Smoker, %**						
Never	42.90 (0.98)	41.04 (1.51)	43.06 (1.69)	42.88 (1.48)	44.20 (1.84)	0.20
Ever	23.66 (0.70)	21.82 (1.05)	22.77 (1.26)	24.63 (1.26)	25.06 (1.50)	0.06
Current	28.64 (0.80)	30.01(1.47)	28.82 (1.30)	28.41 (1.61)	27.63 (1.38)	0.21
**Drinker, %**						
No	55.85 (1.07)	55.37 (1.75)	52.89 (1.77)	56.54 (1.42)	58.38 (1.66)	0.05
Yes	40.24 (1.03)	41.65 (1.69)	42.29 (1.70)	40.56 (1.46)	36.96 (1.58)	0.02
**Dietary data**						
Calcium, mg/day	939.58 (9.87)	961.30 (23.3)	924.81 (16.3)	926.97 (21.9)	947.72 (17.2)	0.78
Protein, gm/day	96.76 (0.77)	99.16 (1.52)	95.95 (1.28)	95.43 (1.62)	96.83 (1.58)	0.39
Energy, kcal/day	2539.65 (15.12)	2590.32 (38.7)	2480.95 (30.5)	2530.37 (32.9)	2563.79 (30.2)	0.98

Data are expressed as weighted means (standard error, Se) or proportions (Se).

**Table 2:** Serum biochemistry and lumbar spine bone mineral density based on SUA quartiles, weighted.

Characteristics	Overall	Quartiles of serum uric acid (mg/dL)				
		Q1 < 5.2	Q2: 5.2–5.9	Q3: 6.0–6.7	Q4 > 6.7	*P* for trend
**Blood laboratory data**						
C-reactive protein, mg/dL	0.36 (0.02)	0.34 (0.04)	0.32 (0.03)	0.34 (0.02)	0.41 (0.03)	0.11
Serum albumin, g/dL	4.42 (0.01)	4.41 (0.01)	4.43 (0.01)	4.43 (0.01)	4.40 (0.01)	0.77
Alkaline phosphatase, U/L	76.16 (0.38)	77.04 (0.93)	75.60 (0.65)	76.26 (0.67)	75.90 (0.70)	0.46
Urea nitrogen, mg/dL	13.59(0.08)	13.39 (0.14)	13.20 (0.12)	13.46 (0.16)	14.22 (0.18)	0.003
Creatinine, mg/dL	0.96 (0.00)	0.90 (0.01)	0.94 (0.01)	0.96 (0.01)	1.02 (0.01)	< 0.001
eGFR, mL/min/1.73 m^2^	106.93 (0.33)	111.67 (0.54)	107.96 (0.49)	106.36 (0.52)	102.82 (0.49)	< 0.001
Serum Calcium, mg/dL	9.53 (0.01)	9.50 (0.01)	9.52 (0.01)	9.56 (0.01)	9.55 (0.01)	0.04
Serum Phosphorus, mg/dL	3.66 (0.01)	3.63 (0.02)	3.66 (0.01)	3.69 (0.02)	3.66 (0.02)	0.24
25-OH-D, ng/mL	21.52 (0.15)	21.74 (0.30)	21.64 (0.28)	21.91 (0.33)	20.88 (0.29)	0.09
Parathyroid hormone, pg/mL	43.44 (0.61)	43.79 (1.33)	42.45 (0.88)	43.05 (1.21)	44.36 (1.67)	0.69
**BMD, gm/cm**^**2**^						
Lumber Spine BMD, gm/cm^2^	1.06 (0.00)	1.06 (0.01)	1.05 (0.01)	1.05 (0.01)	1.06 (0.01)	0.77

Data are expressed as weighted means (standard error, Se) or proportions (Se).

*BMD* Bone Mineral Density, *eGFR* estimated glomerular fltration rate.

### Univariate analysis

Univariate analysis, as shown in Supplementary Table S2, indicated that these factors including age, BMI, serum albumin, serum calcium, serum phosphorus, 1,25D, ALP, dietary energy and protein intake were obviously associated with lumbar spine BMD. However, no associations were detected among the remaining variables.

### Association between SUA and lumbar spine BMD

**Figure 2:**
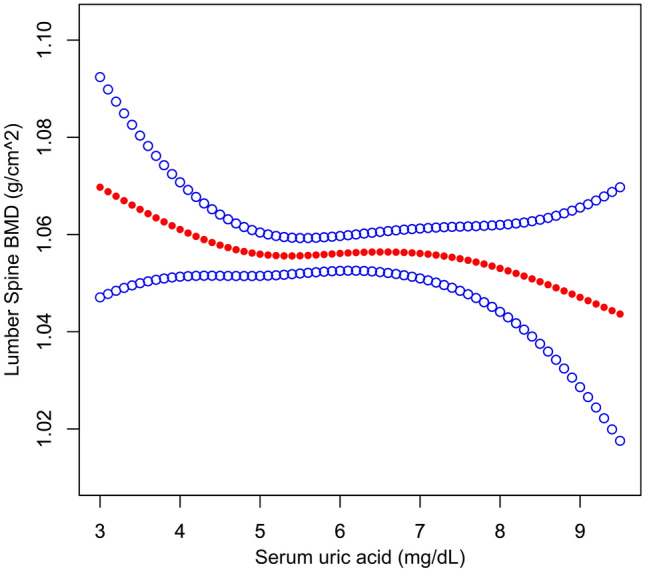
The relationship between serum uric acid and lumbar spine bone mineral density**.** A linear association between them was detected after adjusting for age, race/ethnicity, education, physical activity, smoking, drinking, body mass index, calcium supplementation, energy intake, protein intake, serum calcium, serum phosphorus, 25-OH-D, parathyroid hormone, serum alkaline phosphatase, C-reactive protein, serum albumin and estimated glomerular filtration rate.

Weighted multivariate linear regression models were employed to assess the correlation of SUA with lumbar spine BMD in four distinct models. As shown in Table 3, no statistically significant association was observed between SUA and lumbar spine BMD [β (95% CI), − 0.003 (− 0.007, 0.002)], even after fully adjusting for potential confounding factors. To evaluate the robustness of results, SUA was treated as categorical variables (quartiles and clinical cutoffs) for sensitivity analysis. The general trends were consistent in all models from the lowest quartile group (Q1) to Q4. In four distinct models, taking Q1 as a reference, no differences were detected for lumbar spine BMD across SUA quartiles (all *P* for trend > 0.05). Similar results were obtained based on SUA clinical cutoffs. Besides, as SUA was continuous variable, it is necessary to explore the nonlinear relationship of SUA with lumbar spine BMD. As shown in Fig. 2, we found that the relationship between SUA and lumbar spine BMD was linear (*P* = 0.56 for non-linearity) after fully adjusted potential confounders, which was further verified by the two-piecewise linear regression model (Supplementary Table S3). Moreover, subgroup analyses were further performed to explore other risks, including age, race/ethnicity, physical activity, education, smoking, drinking, BMI, eGFR and survey years, that might influence the relationship of SUA with lumbar BMD. As presented in Table 4, SUA was not statistically associated with lumbar BMD in all stratification analyses. Besides, no interactions were observed based on all priori stratification (all *P* values for interaction > 0.05).

**Table 3:** Association between serum uric acid and lumbar bone mineral density among 6704 US adult males, weighted.

	Model 1	Model 2	Model 3	Model 4
	β (95% CI)	β (95% CI)	β (95% CI)	β (95% CI)
SUA per 1 mg/dL increase	− 0.002 (− 0.006, 0.002)	− 0.002 (− 0.006, 0.002)	− 0.003 (− 0.007, 0.002)	− 0.002 (− 0.007, 0.002)
**SUA clinical cutoffs**				
< 7 mg/dL	Ref	Ref	Ref	Ref
≥ 7 mg/dL	− 0.004 (− 0.015, 0.008)	− 0.005 (− 0.016, 0.007)	− 0.006 (− 0.018, 0.006)	− 0.006 (− 0.018, 0.007)
**SUA (quartile)**				
Q1	Ref	Ref	Ref	Ref
Q2	− 0.007 (− 0.021, 0.007)	− 0.007 (− 0.021, 0.006)	− 0.006 (− 0.020, 0.008)	− 0.006 (− 0.020, 0.009)
Q3	− 0.004 (− 0.021, 0.013)	− 0.005 (− 0.022, 0.012)	− 0.004 (− 0.020, 0.013)	− 0.004 (− 0.021, 0.013)
Q4	− 0.004 (− 0.018, 0.011)	− 0.005 (− 0.020, 0.009)	− 0.005 (− 0.020, 0.010)	− 0.004 (− 0.020, 0.011)
*P* for trend	0.774	0.605	0.650	0.696

Model 1 was adjusted for none. Model 2 was adjusted for age, race/ethnicity, education, physical activity, smoking and drinking. Model 3 was adjusted for all covariables in model 2 plus body mass index (BMI), calcium supplementation, energy intake, protein intake, serum calcium, serum phosphorus, 25-OH-D (1,25,D), parathyroid hormone, serum alkaline phosphatase (ALP), C-reactive protein (CRP), serum albumin and estimated glomerular filtration rate (eGFR). Model 4 was adjusted for age, race/ethnicity, smoking, BMI, serum albumin, eGFR, CRP, serum calcium, 1,25,D and calcium supplementation. The adjusted variables in model 4 were determined based on the change in effect of more than 10% when added to this model.

**Table 4:** Subgroup analyses of the association of serum uric acid with lumbar bone mineral density, weighted.

Characteristics	N, unweighted	Lumber spine BMD	*P*-value	*P* for interaction
		β (95%CI)		
**Age, years**				0.53
< 40	3475	− 0.005 (− 0.011, 0.001)	0.109	
≥ 40, < 60	1956	0.001 (− 0.006, 0.008)	0.781	
≥ 60	1273	− 0.004 (− 0.013, 0.006)	0.452	
**Race/Ethnicity**				0.75
Non‐Hispanic White	3032	− 0.003 (− 0.008, 0.003)	0.384	
Non‐Hispanic Black	1436	0.002 (− 0.005, 0.009)	0.572	
Mexican American	1708	− 0.004 (− 0.011, 0.003)	0.279	
Other race	528	− 0.006 (− 0.019, 0.007)	0.371	
**Education**				0.76
< High school	2186	− 0.003 (− 0.009, 0.003)	0.256	
High school	1653	− 0.002 (− 0.009, 0.005)	0.662	
> High school	2856	− 0.004 (− 0.009, 0.002)	0.203	
**Physical activity**				0.16
Sedentary	1194	− 0.006 (− 0.014, 0.002)	0.115	
Low	1586	0.001 (− 0.006, 0.008)	0.804	
Moderate	1058	0.004 (− 0.006, 0.013)	0.431	
High	2173	− 0.006 (− 0.012, 0.000)	0.048	
**Smoker**				0.59
Never	2530	− 0.003 (− 0.009, 0.003)	0.299	
Ever	1514	0.002 (− 0.005, 0.009)	0.657	
Current	1698	− 0.007 (− 0.014, 0.000)	0.055	
**Drinker**				0.54
No	4002	− 0.002 (− 0.007, 0.002)	0.315	
Yes	2408	− 0.001 (− 0.006, 0.005)	0.829	
**BMI, kg/m**^**2**^				0.39
< 24	1887	− 0.005 (− 0.012, 0.002)	0.149	
≥ 24, < 28	2208	0.000 (− 0.006, 0.006)	0.959	
≥ 28	2609	− 0.002 (− 0.007, 0.003)	0.492	
**eGFR, mL/min/1.73 m**^**2**^				0.21
< 90	1072	− 0.005 (− 0.013, 0.004)	0.235	
≥ 90, < 120	3855	− 0.003 (− 0.008, 0.001)	0.149	
≥ 120	1777	0.004 (− 0.003, 0.010)	0.269	
**Survey years**				0.64
1999–2000	1595	− 0.002 (− 0.010, 0.005)	0.548	
2001–2002	1781	0.001 (− 0.006, 0.008)	0.847	
2003–2004	1742	− 0.003 (− 0.010, 0.002)	0.174	
2005–2006	1586	− 0.003 (− 0.010, 0.004)	0.428	

The model was adjusted for age, race/ethnicity, education, physical activity, smoking, drinking, body mass index, calcium supplementation, energy intake, protein intake, serum calcium, serum phosphorus, 25-OH-D, parathyroid hormone (PTH) serum alkaline phosphatase (ALP), C-reactive protein (CRP), serum albumin and estimated glomerular filtration rate except the corresponding stratification variable.

Serum ALP, PTH and CRP were log2 transformed prior to regression analysis.

## Discussion

In this nationally representative population of US adult men, SUA was not statistically significantly associated with lumbar spine BMD. Besides, this association was independent of other factors related to bone health, such as age, race/ethnicity, dietary factors, BMI, eGFR, serum calcium, serum phosphorus, 1,25D, and PTH. Moreover, results remained consistent in all stratified analyses, even in a seemingly unfavorable condition, including advanced age, race and obesity. Correspondingly, none of priori stratifications modified the association of SUA with lumbar spine BMD (*P* for interaction > 0.05).

Increasing evidence indicated that elevated SUA levels were related to higher risk of various adverse outcomes including progressive renal disease, metabolic syndrome, diabetes mellitus, cardiovascular disease and stroke^7–12^. In contrast with these potential deleterious effects, higher SUA levels might protect against bone loss probably via the potential anti-oxidant effects^13–17^. However, observational data on the relationship of SUA with lumbar spine BMD have been mixed. Four Chinese cross-sectional studies that included postmenopausal women and older adults revealed a positive association of a higher SUA with greater BMD^19,33–35^, with similar findings among other studies from Asia^13,14,36,37^. In contrast, another Chinese cross-sectional study showed a positive association of SUA with lumbar spine BMD only in postmenopausal women (n = 4256), rather than in men (n = 943)^20^. Similarly, a population-based cross-sectional study in US of 6759 participants over 30 years demonstrated no association was observed between SUA and lumbar spine BMD, and this was further confirmed in experimental hyperuricemia rats^23^. Moreover, a Mendelian randomized analysis of 1108 postmenopausal women and 226 older men in China reported that no causal effect of SUA on BMD was found measured at various sites^38^. Taken together, similar to previous observational studies^20,23^, our findings covered men aged 18–85 years did not provide an inspiring clue for the hypothesis that SUA might be beneficial to lumbar spine BMD.

The potential causes for discrepancy between prior studies might be due to different population characteristics (such as race, age, and BMI), or differences of potential confounders adjusted for. Firstly, the positive association of SUA with BMD was mainly in Asian populations^13–15,33–37^, but not in US populations^23^, similar to the present study on US adult men. Notably, when we further stratified by race, no difference was found between different races in the US population. Thus, whether this difference exists only in Eastern and Western populations requires further research. Secondly, the favorable correlation of UA with lumbar spine BMD was reported mostly among peri- and postmenopausal women or elderly men^13–15,33,34,36^, while the present study focused on the general male population with a larger age span (18–85 years), and no correlation was observed between SUA and lumbar spine BMD. However, when we stratified by age, no association was found even in subjects ≥ 60 years old, and no age-modification effect was observed. Thirdly, a cross-sectional study from South Korea detected effect modification by BMI, and observed a stronger association of SUA with lumbar BMD only in non-obese individuals but not in obese individuals^21^. However, we did not detect effect modification by BMI, which was consistent with previous study by Muka et al.^39^. Currently, the effect of obesity on BMD remains controversial. Nabipour et al.^40^ indicated a positive correlation of BMI with BMD in older men, while Cervellati et al.^41^ reported an inverse association in postmenopausal women. Fourthly, most importantly, evidence from many observational studies failed to fully control potential confounders, such as serum calcium, serum phosphorus, ALP, 1,25D or PTH^13,15,19–21^, all of which have been linked to bone metabolism. Finally, more evidence supporting the positive correlation of SUA with BMD may be partly due to publication bias, while the “negative” results are more likely to be underestimated.

In fact, related studies focused on the mechanism of SUA on BMD are paradoxical. The benefits of SUA for bone health are mainly based on the fact that UA may exert antioxidative properties that can prevent oxidative stress-related bone loss in osteoporosis. However, the antioxidant properties of UA mainly act in human plasma and may be interfered by the hydrophobic lipid layer of the cell membrane^42,43^. Moreover, intracellular free oxygen radicals are generated during UA degradation, which further enhances intracellular superoxide generation by interacting with NADPH oxidase^44^, thereby inhibiting osteoblast bone formation and stimulating osteoclast bone resorption^45,46^. Additionally, SUA might exert adverse effects on bone health by affecting 1,25D and PTH levels. Several studies have emphasized the inverse correlation between 1,25D concentrations and SUA in hyperuricemic rats^47^ and in patients with CKD^48,49^, and this association could be reversed after receiving allopurinol to lower SUA levels^49^. Likewise, two observational studies showed that SUA was positively correlated with PTH levels^47,50^. In brief, the correlation of SUA with BMD remains complicated and controversial. Future more prospective studies are required to clarify a clearer relationship between them.

The strength of this study, rigorous inclusion criteria and thorough adjusting for potential confounding factors, presented a lack of association of SUA with lumbar spine BMD in this nationally-representative sample of US adult males, and further expanded the evidence for the SUA-BMD association in different populations and races. However, a few limitations need to be acknowledged. First, a cross-sectional study design tends to only constrain to assessing associations but uncertainty concerning the temporal relationship of exposure–outcome. Therefore, further prospective studies and basic mechanistic research are vital to clarify the exact effect SUA on lumbar spine BMD. Second, questionnaire surveys were used to collect some data such as dietary factors, smoking, drinking and sports activities, which inevitably had some recall bias. Third, since the NAHANS database only provided prescription drug usage within one month before the survey date, we only removed individuals who used drugs affecting bone metabolism or SUA, but failed to take the medicine history into consideration. Therefore, the influence of drug residue may still exist, especially bisphosphonates and glucocorticoids. Fourth, the participants were restricted to US adult men. Thus, these findings could not be generalized to non-adult men, women, or other races/ethnicities. Finally, despite adjusting for potential known confounding factors, residual or unmeasured confounder remains possible.

In summary, this study demonstrated that SUA levels were not associated with lumbar spine BMD in this nationally representative population of US adult males, even fully adjusting for confounding variables. Due to the lack of causal association evidence, further prospective studies and basic mechanistic research are needed to clarify the exact effect of SUA on lumbar spine BMD.

## Supplementary Information

Supplementary Information.

## Data Availability

The datasets are available on https://doi.org/10.5061/dryad.d5h62.
