# Supplementary material for: No association between serum uric acid and lumbar spine bone mineral density in US adult males: a cross sectional study
Source: Sci Rep. 2021 Aug 2;11:15588. doi: 10.1038/s41598-021-95207-z (PMC8329127; doi:10.1038/s41598-021-95207-z)
Supplement: Supplementary file 1 — Supplementary Information. [file 41598_2021_95207_MOESM1_ESM.pdf]

No association between Serum Uric Acid and Lumbar Spine Bone Mineral Density in US adult males: a cross sectional study

Xiaoli Li[1,2,*], Lianju Li[1], Lixian Yang[3], Jiaxun Yang[4], & Hua Lu[5]

[1]Department of Rheumatology, Xingtai people's Hospital, Xingtai 054001, Hebei, China

[2]Department of Internal Medicine, Hebei Medical University, Shijiazhuang 050017, Hebei, China.

[3]Department of breast surgery, Xingtai people's Hospital, Xingtai 054001, Hebei, China

[4]Department of Information Center, Xingtai people's Hospital, Xingtai 054001, Hebei, China

[5]Department of Nephrology, Xingtai people's Hospital, Xingtai 054001, Hebei, China

***Corresponding author:**

Xiaoli Li, Email: drlixiaoli86@163.com

Supplementary Information

Supplementary Table S1. Description of the missing variables

Variables	No.	Missing data, N
Age	6704	0
Race	6704	0
Education	6695	9
Physical activity	6011	693
Smoking	5742	962
Drinking	6410	294
Weight	6677	27
Height	6666	38
BMI	6656	48
CRP	6702	2
Serum Albumin	6704	0
ALP	6704	0
BUN	6704	0
Scr	6704	0
eGFR	6704	0
Serum Calcium	6704	0
Serum Phosphorus	6704	0
25-OH-D	5121	1583
PTH	3285	3419
Calcium intake	6423	281
Dietary energy intake	6423	281
Dietary protein intake	6423	281
Lumber Spine BMD	6704	0

Abbreviations: *ALP* Alkaline phosphatase, *BMD* Bone Mineral Density, *BMI* Body mass index, *BUN* Blood urea nitrogen, *CRP* C-reactive protein, *eGFR* estimated glomerular fltration rate, *PTH* Parathyroid hormone, *Scr*, Serum creatinine

Supplementary Table S2. The results of univariate analysis, weighted.

Characteristics	Statistics	Lumber Spine BMD β (95% CI)	*P*
Age, years	40.49 (0.27)	0.000 (0.000, 0.001)	0.031
Race, %			
Non-Hispanic White	70.65 (1.41)	Ref	
Non-Hispanic Black	10.05 (0.82)	-0.009 (-0.021, 0.001)	0.093
Mexican American	9.26 (0.81)	0.001 (-0.008, 0.011)	0.817
Other race	10.04 (1.00)	-0.002 (-0.019, 0.014)	0.752
Education, %			
< High school	19.52 (0.79)	Ref	
High school	26.28 (0.86)	-0.003 (-0.017, 0.010)	0.590
> High school	54.05 (1.15)	-0.004 (-0.016, 0.009)	0.566
Physical Activity, %			
Sedentary	12.94 (0.65)	Ref	
Low	24.18 (0.71)	-0.008 (-0.023, 0.008)	0.328
Moderate	17.94 (0.64)	-0.004 (-0.022, 0.014)	0.672
High	34.73 (0.78)	-0.006 (-0.022, 0.010)	0.473
Smoking status, %			
Never	42.90 (0.98)	Ref	
Past	23.66 (0.70)	-0.007 (-0.021, 0.005)	0.238
Current	28.64 (0.80)	-0.010 (-0.023, 0.002)	0.116
Drinking status, %			
No	55.85 (1.07)	Ref	
Yes	40.24 (1.03)	-0.002 (-0.013, 0.009)	0.677
BMI, kg/m^2	27.55 (0.10)	0.001 (0.000, 0.002)	0.030
Log_2 (CRP, mg/dL)	-2.74 (0.03)	0.001 (-0.002, 0.003)	0.587
Serum Albumin, g/dL	4.42 (0.01)	0.007 (0.001, 0.019)	0.007
Log_2 (ALP, U/L)	6.18 (0.01)	0.012 (0.001, 0.022)	0.028
eGFR, ml/min/1.73m^2	106.93 (0.33)	0.000 (-0.000, 0.000)	0.421
Serum Calcium, mg/dL	9.53 (0.01)	-0.021 (-0.033, -0.008)	<0.001
Serum Phosphorus, mg/dL	3.66 (0.01)	-0.005 (-0.008, -0.002)	0.012
25-OH-D, ng/mL	21.52 (0.15)	-0.001 (-0.001, -0.000)	<0.001
Log_2 (PTH, pg/mL)	5.28 (0.01)	0.002 (-0.008, 0.012)	0.287
Calcium intake, mg/day	939.58 (9.87)	0.000 (0.000, 0.000)	0.303
Energy intake, kcal/day	2539.65 (15.12)	0.000 (0.000, 0.000)	0.019
Protein intake, g/day	96.76 (0.77)	0.000 (0.000, 0.000)	0.046

Statistics data are expressed as weighted means (standard error, Se) or proportions (Se).

Abbreviations: *ALP* Alkaline phosphatase, *BMD* Bone Mineral Density, *BMI* Body mass index, *CI,* confidence interval, *CRP* C-reactive protein, *eGFR* estimated glomerular fltration rate, *PTH* Parathyroid hormone, *Ref* reference

Supplementary Table S3. The result of two-piecewise linear regression model

	Lumber Spine BMD (gm/cm^2)	
	β (95% CI)	*P*
Fitting model by standard linear regression	-0.003 (-0.007, 0.002)	0.318
Fitting model by two-piecewise linear regression<br>Inflection point of SUA 4.3 mg/dL		
≤ 4.3 mg/dL	-0.023 (-0.052, 0.005)	0.109
>4.3 mg/dL	-0.001 (-0.004, 0.003)	0.760
P for log likelihood ratio test		0.135

The model was adjusted for age, race / ethnicity, education, physical activity, smoking, drinking, body mass index, calcium supplementation, energy intake, protein intake, serum calcium, serum phosphorus, 25-OH-D, parathyroid hormone, serum alkaline phosphatase, C-reactive protein, serum albumin and estimated glomerular filtration rate.
